# Mortality, functional outcomes and quality of life after hip fractures complicated by infection: a case control study

**DOI:** 10.5194/jbji-6-347-2021

**Published:** 2021-09-23

**Authors:** Antonios A. Koutalos, Christos Baltas, Vasileios Akrivos, Christina Arnaoutoglou, Konstantinos N. Malizos

**Affiliations:** Department of Orthopaedic Surgery & Musculoskeletal Trauma, Faculty of Medicine, University of Thessaly, Larissa, Greece

## Abstract

**Introduction**: Infection is a detrimental complication of operatively treated hip fractures. The objective of this retrospective case-control study was to evaluate the mortality, the physical function and the quality of life of hip fractures complicated with infection and determine risk factors for deep infection in hip fractures. **Patients and methods**: All patients with hip fractures (31A and 31B OTA/AO) that were operatively managed over a 10-year period that subsequently developed deep infection were included in the study. Thirty-nine patients met the inclusion criteria. These patients were compared with a matched control group of 198 patients without infection. Minimum follow-up was 1 year. Mortality, Barthel index score, EQ-5D-5L, Parker mobility score and visual analogue scale (VAS) pain score were compared between groups. **Results**: Mortality at 1 month was 20.5 % and 43 % at 1 year. Half of the infections were acute and 28 % were polymicrobial. Mortality was greater in the infection group (43 % vs. 16.5 %, p<0.0014), and Barthel index was inferior in the infection group (14 vs. 18, p<0.0017) compared to control group. Logistic regression analysis revealed that time from admission to surgery was a negative factor that predisposed to infection. **Conclusions**: Patients complicated with infection after a hip fracture have higher mortality and inferior functional results. Delay from admission to surgery predisposes to infection.

## 1 Introduction

Fragility hip fractures are associated with significant limitation in physical activity and mortality among the elderly patients (Johnell and Kanis, 2004). In addition to this, infection is a dreadful complication with negative effects on function and mortality (Partanen et al., 2006). The incidence of fracture-related infection (FRI) after hip fixation surgery is reported between 1.2 % and 3.6 % (Pollard et al., 2006; Edwards et al., 2008). Diabetes mellitus, steroids treatment and operative time have been shown by several studies to increase the risk of infection (Partanen et al., 2006; Edwards et al., 2008).

The FRIs after hip fractures are devastating for the patient and their family, require multiple operations, antibiotics, increased length of stay (LoS) in the hospital, office visits and much longer rehabilitation (Pollard et al., 2006; Edwards et al., 2008). The current protocols involve patient optimisation of the general health and nutritional status, surgical management without delay, control of blood loss and early mobilisation after hip fracture fixation, to diminish the risk of complications and infection.

The purpose of the study was (1) to explore any risk factors that predispose to deep infection after hip fracture surgery, (2) to examine the impact of infection on mortality, and (3) to evaluate the final functional outcomes. The hypothesis is that patients with infections would have higher mortality and lower functional status.

## 2 Patients and methods

This was a case control study of geriatric patients with hip fractures that underwent surgical management and were complicated by infection. From 2010 to 2020, 5453 hip fractures were surgically managed in a teaching centre. In the same period 39 patients were complicated with FRI and were treated accordingly. Inclusion criteria included cases with deep infection, diagnosed after operative fixation of a hip fracture (31A and 31B according to OTA/AO classification) and a minimum follow-up of 1 year.

Exclusion criteria were hip surgery performed for reasons other than fracture, patients treated for the hip fracture in other hospitals and were referred to our tertiary centre for infection management, hip fractures that were managed conservatively, and superficial infections or skin ulcers. Patients younger than 65 were also excluded because the main focus of our study was elderly patients having low-energy hip fractures. These patients were compared with a subset of patients that had hip fracture and have been part of the prospectively maintained local database since 2018. Epidemiological, clinical, radiographic, and pre- and postoperative functional scores are stored in the intra-departmental database.

**Table 1:** Demographics of the group of patients that complicated with infection after fracture fixation.

Sex	
Male [n (%)]	8 (21 %)
Female [n (%)]	31 (79 %)
Age [mean (SD)]	79 (13)
Smoking	
Yes [n (%)]	8 (21 %)
No [n (%)]	31 (79 %)
Accommodation	
Home [n (%)]	37 (95 %)
Not living in own home [n (%)]	2 (5 %)
Dementia	
Yes [n (%)]	5 (13 %)
No [n (%)]	34 (87 %)
Diabetes mellitus	
Yes [n (%)]	11 (28 %)
No [n (%)]	28 (72 %)
Mobilisation without assistance	
Yes [n (%)]	33 (85 %)
No [n (%)]	6 (15 %)
Use of anti-coagulants	
Yes [n (%)]	21 (54 %)
No [n (%)]	18 (46 %)
Haemoglobin at admission [mean (SD)]	11.5 (2.4)
Albumin at admission [mean (SD)]	3.6 (0.8)
ASA score [mean (SD)]	2.5 (0.2)
Fracture type	
Extra-capsular [n (%)]	25 (64 %)
Intra-capsular [n (%)]	14 (36 %)
Length of stay on first admission (days) (LOS) [median (IQR)]	12 (31)
Length of stay combining all admissions (days) (LOS) (mean, SD)	37 (8)
Operative time (hours) [median (IQR)]	1.4 (0.6)
Number of operating room visits for debridement [median (IQR)]	2 (2)
Fate of implants	
Removed [n (%)]	16 (41 %)
Not removed [n (%)]	23 (59 %)

Diagnosis of infection was established if two or more deep intraoperative cultures were positive, in the presence of a sinus communicating with the hip, in the occurrence of wound breakdown or the presence of pus coming beneath the fascia (Metsemakers et al., 2018).

A management plan was agreed on by the multidisciplinary team composed of an orthropaedic surgeon, an infection disease specialist and a geriatrician/internal medicine specialist. Host optimisation involved correction of malnutrition and hyperglycemia if present. Serial debridement, antibiotics and implant retention (DAIR), antibiotic-loaded bone cement spacers (ALBCS) and vacuum-assisted closure (VAC) devices were implemented to eliminate infection, together with IV antibiotics for 6 weeks. An attempt was made for implant retention in the acute infections (diagnosed <6 weeks) until fracture healing occurred. On the contrary, implants were removed in late infections (diagnosed ≥6 weeks) with healed fracture, in unstable implants or fractures, and in immunocompromised patients (host C according to Cierny) (Cierny et al., 2003). Follow-up after discharge included outpatient clinic visits at 3, 6 and 12 weeks for clinical examination and radiographs if needed and then annually outpatient visits.

Demographics were recorded (Table 1). Patient information, including smoking, accommodation place involving geriatric centres or own residence, the presence of dementia, diabetes mellitus, if they walked with or without assistance, the use of anti-coagulants, haemoglobin and albumin levels at admission, was retrieved from the medical notes. Pre-operative function was assessed with the Barthel index.

Data regarding fracture type (extra- or intra-capsular), type of surgery performed, length of stay in the first admission, total length of stay combining all admissions, ASA score (Owens et al., 1978), delay till the operation after admission, mean operative time, number of debridements and if implants were finally removed or not were collected from the medical records. Complications during hospital stay were also recorded. Information on 30-day and 1-year mortality, the length of follow-up, the time of infection diagnosis from operation, the bacteria responsible for the infection, Gram stain, and mono- or polymicrobial infection was also documented.

In the last follow-up, patients were interviewed at the outpatient clinic or over telephone questionnaire by a researcher not involved in their management. Visual analogue scale (VAS) pain score, Parker mobility score (Parker and Palmer, 1993), Barthel index (Mahoney and Barthel, 1965) and EQ-5D-5L (Herdman et al., 2011) utility index were assessed. Parker mobility measures the mobility of the patient indoors, outdoors and during shopping and is often used as an outcome measure in hip fractures or as a predictor of mortality (Kristensen et al., 2010). Barthel index evaluates the ability to perform basic activities of daily living and has been widely utilised in geriatric patients. It has been validated in hip fractures (Bouwstra et al., 2019; Mayoral et al., 2019). Minimal clinical important difference is calculated to three points (Xu et al., 2019). EQ-5D-5L measures the quality of life with a utility index ranging from 0 (death) to 1 (perfect health). The average utility after hip fracture varies from 0.379 at 4 months to 0.67 at 24 months (Polinder et al., 2007; Sims et al., 2018). The minimum clinical important difference is 0.08 (Walters and Brazier, 2005).

### 2.1 Control group

The control group consisted of 210 consecutive patients with hip fractures operated from January to June 2018. Twelve patients were lost to follow-up. Minimum follow-up was 1 year. The same demographics, clinical data and information regarding the fracture, the surgery and complications are recorded in these patients. Pre- and postoperative Barthel index score was documented. Postoperative functional level evaluation including Parker mobility score and quality of life data involving EQ-5D-5L index are also available for these patients. Mortality at 1 month and at 1 year was also calculated.

### 2.2 Outcomes

The primary outcome was the mortality rate and the functional outcome as measured with the Barthel index score. Secondary analysis included the examination of whether infection influences the final Barthel index score and the search for predisposing factors of infection after hip fractures.

### 2.3 Statistics

Descriptive statistics were used for demographics. Normality of the data was evaluated with the Shapiro–Wilk test. For normally distributed data, mean ± SD was presented, while for skewed data, median ± interquartile range (IQR) was used. T test was utilised for comparing groups of normally distributed data and the non-parametric Mann–Whitney U test otherwise. Chi-squared test was used for categorical data analysis. Regression analysis was used for testing the hypothesis of whether infection had an effect on Barthel index score. Except infection, the variables tested included age, sex, smoking, dementia, diabetes mellitus, previous accommodation, previous mobilisation status, use of anti-coagulants, haemoglobin, albumin, ASA score, fracture type, method of fixation, length of stay, operative time and waiting time until surgery. Finally, logistic regression analysis was employed for the search of any predisposing factors to infection among the same variables. SPSS statistics package v.25 (IBM, Armonk, New York, USA) was used for statistical analyses.

## 3 Results

From 2010 to 2020, 5453 hip fractures received surgical treatment, of which 3435 were extra-capsular and 2018 were intra-capsular fractures (31A and 31B OTA/AO classification respectively). The infection rate over the entire period of observation was 0.72 % (39 of 5453 hip fractures). Extra-capsular fractures were complicated by infection in 0.69 % of the cases (14 of 2018) and intra-capsular fractures in 0.72 % (25 of 3435). The rate of infection after hemiarthroplasty for the intra-capsular fractures was 0.7 %, in the patients fixed with intramedullary nails 0.8 % and 0.6 % in patients with sliding hip screws. No infections were diagnosed after total hip arthroplasty or cannulated screws. Twenty infections were diagnosed from 2010 to 2014 and 19 from 2015 to 2020. Approximately half of the infections (20 of 39) were diagnosed in less than 6 weeks. The patients underwent a mean of two surgical debridement for eradication of the infection (range 1–5). With regard to the microbiology of the infection, gram-positive micro-organisms were isolated in 48 % of cases with methicillin-resistant *Staphylococcus aureus* (MRSA) being most common. Polymicrobial infections consisted 28 % of the cases. In one case, no bacteria were identified (Table 2).

**Table 2:** Micro-organisms isolated from infected hip fracture cases.

Micro-organism isolated	Frequency [n (%)]
Gram positive	
*Staphylococcus aureus* MSSA	6 (15 %)
*Staphylococcus aureus* MRSA	7 (18%)
*Staphylococcus epidermidis*	6 (15 %)
Gram negative	
*Escherichia coli*	3 (8 %)
*Acinetobacter baumannii*	5 (13 %)
Polymicrobial infections	11 (28 %)
*Enterococcus faecalis*+*Acinetobacter baumannii*	4 (10 %)
*Staphylococcus aureus* MRSA +*Pseudomonas aeruginosa*	3 (7.5%)
*Staphylococcus aureus* MRSA +*Klebsiella pneumoniae*	3 (7.5 %)
*Staphylococcus aureus* MRSA +*Candida* spp.	1 (3 %)
Negative cultures	1 (3 %)

**Table 3:** Comparison between hip fracture patients with infection and without infection. Mann–Whitney U test was used for continuous variables and chi square for categorical.

	Infection group	Control group	p value
Barthel index score pre-op [(median (IQR)]	20 (1)	20 (1)	0.872
Barthel index score final [(median (IQR)]	14 (16)	18 (5)	0.017*
EQ-5D-5L index [(median (IQR)]	0.742 (0.75)	0.769 (0.123)	0.065
Parker mobility score [(median (IQR)]	8 (4)	8 (3)	0.071
VAS pain score [(median (IQR)]	1 (1)	0 (2)	0.107
Mortality at 30 d [n (%)]	8 (20.5 %)	8 (4.0 %)	0.016*
Mortality at 1 year [n (%)]	17 (44 %)	29 (14.6 %)	0.014*

* p<0.05

Eight patients died after the infection within the first month and 17 patients at 1 year (21.5 % and 43 % respectively). Two patients died while in hospital, and their death was attributed to infection. In comparison, in the control group the mortality rate was 4.0 % at 1 month and 14.6 % at 1 year. This was statistically different in both cases (chi square, p=0.016 and p=0.014 respectively) (Table 3). At the final follow-up, two patients were not traced and four more died after the first year, leaving 16 patients for the final evaluation. For those patients who were available the mean follow-up was 4.5 years (range 1.5 to 9.5 years). At the final follow-up VAS, Parker, Barthel and EQ-5D-5L were measured (Table 3). The control group had better functional score as measured with the Barthel index, and this difference was both statistically and clinically significant. The control group had better mobility status (Parker mobility score), less pain (VAS pain score) and better quality of life (EQ-5D-5L), but these differences did not reach significance (Table 3). Complications were more common in the infection group (41 % vs. 8.3 %) (chi-squared test, p<0.001) and included lung infections, urinary tract infections, strokes, myocardial infractions, deep vein thrombosis and acute kidney injuries.

**Table 4:** List of variables tested in the model regarding the final Barthel index score. Variables with statistically significant correlations or differences were entered in the multiple regression model.

Univariate analysis					
	Value (Barthel index score)		p value		
Sex (female/male) (mean ± SD)	16±6 vs. 17.5±4		0.09		
Smoking (yes/no) (mean ± SD)	17.4±6 vs. 15.9±5		0.954		
Diabetes mellitus (yes/no) (mean ± SD)	15.3±6 vs. 16.7±5		0.303		
Dementia (yes/no) (mean ± SD)	12±7 vs. 17±4		0.017		
Previous accommodation (home/geriatric centre) (mean ± SD)	16.8±5 vs. 13.1±8		0.056		
Previous mobilisation status (independent/with assistance) (mean ± SD)	17.2±5 vs. 15±5.5		0.275		
Use of anti-coagulants (yes/no) (mean ± SD)	15.7±5 vs. 16.5±6		0.649		
Fracture type (extra-/intra-capsular) (mean ± SD)	17.3±4 vs. 15.2±6		0.064		
Infection (yes/no) (mean ± SD)	14.1±8 vs. 17.6±5		0.014		
Age	Pearson correlation (-0.326)		0.009		
Operative time	Pearson correlation (0.246)		0.063		
Time from admission to surgery	Pearson correlation (-0.245)		0.073		
ASA score	Pearson correlation (-0.165)		0.212		
Haemoglobin at admission	Pearson correlation (0.314)		0.023		
Albumin at admission	Pearson correlation (0.089)		0.541		
Pre-operative Barthel index score	Pearson correlation (0.703)		<0.001		
Length of stay	Pearson correlation (-0.310)		0.029		
Multivariate analysis model					
Model	Unstandardised coefficients		Standardised coefficients	t	Significance
	B	Standard error	Beta		
(Constant)	0.850	6.590		0.129	0.898
Age	-0.009	0.073	-0.012	-0.123	0.902
Infection	-1.012	0.445	-0.231	-2.276	0.027
Pre-operative Barthel score	1.038	0.165	0.639	6.303	<0.001
Type of fracture	0.757	0.535	0.133	1.416	0.163
Dementia	0.558	1.560	0.035	0.358	0.722

**Table 5:** Comparison different pre-operative, operative and postoperative factors between infection and control group that used for the logistic regression model to explore if any of them predisposed to infection.

	Infection group		Control group		Univariate analysis (p value)	
Age [mean (SD)]	79 (13)		81 (8)		0.511	
Male sex [n (%)]	8 (21 %)		49 (25 %)		0.775	
Smoking [n (%)]	8 (21 %)		18 (9 %)		0.185	
Accommodation [n (%)]	37 (95 %)		190 (96 %)		0.876	
Dementia [n (%)]	5 (13 %)		38 (19 %)		0.554	
Diabetes mellitus [n (%)]	11 (28 %)		53 (27 %)		0.729	
Mobilisation without assistance [n (%)]	33 (85 %)		147 (74 %)		0.360	
Use of anti-coagulants [n (%)]	21 (54 %)		93 (47 %)		0.654	
Wound oozing [n (%)]	14 (36 %)		18 (9 %)		0.007*	
Haemoglobin at admission [mean (SD)]	11.5 (2.4)		11.8 (1.9)		0.760	
Albumin at admission [mean (SD)]	3.6 (0.8)		3.5 (0.5)		0.779	
ASA score [mean (SD)]	2.5 (0.7)		2.3 (0.6)		0.411	
Anesthesia (general) [n (%)]	8 (20 %)		12 (6 %)		0.094	
Fracture type (extra-capsular) [n (%)]	25 (64 %)		91 (46 %)		0.039*	
Operative time (hours) [median (IQR)]	1.5 (0.6)		1 (0.42)		0.038*	
Time to surgery (days) [median (IQR)]	6 (5)		2.3 (2)		0.001*	
Multivariate analysis model						
Variables	B	Standard error	Significance	Exp(B)	95 % CI for Exp(B)	
					Lower	Upper
Operative time	-1.308	0.826	0.114	0.412	0.038	4.423
Time from admission to surgery	-0.597	0.261	0.022	0.551	0.330	0.918
fracture type	19.573	6665	0.998	327 812 189	–	–
Wound oozing	0.865	1.217	0.477	2.374	0.219	25.766
Constant	4.382	1.756	0.013	80.027		

* p<0.05

The final functional outcome as measured with Barthel index score was affected by the previous functional state (Barthel index before hip fracture), haemoglobin at admission, age, time from admission to surgery, operative time, and the presence of dementia and infection (univariate analysis). Besides pre-fracture functional outcome, the multiple regression model showed that infection (p=0.03) independently affected the final Barthel score. The model explained the 70 % of the variability of the final functional outcome (Table 4). Finally, a logistic regression model was utilised to identify any risk factors that predisposed to infection. While increased operative time, time from admission to surgery, extra-capsular fracture and wound oozing at 7 d were found to increase the risk of infection, in the multivariate analysis only time to surgery remained a significant risk factor (p=0.022) (Table 5). The model predicted that every day of delay increases the odds ratio of infection by a factor of 1.8.

## 4 Discussion

In the present study, the infection after hip fracture in elderly patients was associated with diminished functional ability and higher mortality. This study also demonstrated that the final functional outcome of patients with hip fractures depends on the preoperative physical function and is negatively affected by the presence of infection. Delay in surgery almost doubles the risk for infection. Even if the incidence of infection is rather small, given the frequency of geriatric hip fractures and the poor outcomes of infection management, every attempt should be made to avoid this devastating complication.

The 1-year mortality in the FRI group was 44 % compared to 14.6 % in the control group. Duckworth et al. (2012) also found higher mortality rate in patients with deep infection after hip fractures compared to patients without infections, although not statistically significant (33 % vs. 28 %). In the same study it was reported that dementia and *S. aureus* infection were associated with increased mortality.

The rate of infection in this series was 0.72 %, lower than the reported range of 1.2 % to 3.6 % (Pollard et al., 2006; Edwards et al., 2008). The most common causative micro-organism is *Staphylococcus aureus* or *Staphylococcus epidermidis*, similar to the literature reports (Partanen et al., 2006; Edwards et al., 2008). Factors predisposing to fracture-related infection after geriatric hip fractures are numerous and involve diabetes mellitus (Partanen et al., 2006), oral steroids (Edwards et al., 2008) and very long operative time (>240 min) (Edwards et al., 2008). In another recent study the rate of superficial site infection (SSI) after hip fractures reached 3.67 %, and diabetes, summer period, steroids therapy, increased BMI and anaemia were recognised as risk factors (Ji et al., 2019). In the current study, we found that increased operative time, delays from admission to surgery, extra-capsular fracture and wound leakage in the first 7 d increased the probability of infection in the univariate analysis. However, the multivariate model disclosed time from admission to surgery as the only statistically significant risk factor. This, to our knowledge, has been described in the literature for the first time. This time period does not include the time from fracture to admission, which is usually within hours after the fall causing the fracture. Given the fact that the period time from admission to surgery is a modifiable factor, every attempt should be made to shorten the time to surgery. It could be argued that the use of anticoagulants or multiple co-morbidities could be confounding factors for increased likelihood of infection as they could delay the time to fracture fixation. However, the frequency of anticoagulants and the ASA score, which is a proxy for comorbidity, were comparable between the two groups. Year of infection could also be a confounding factor as the management of hip fractures differs throughout the years. Still, we observed that half of the infections occurred from 2010 to 2014 and half from 2015 to 2020, while the number of hip fractures remained stable. A longer preoperative hospitalisation may be followed by colonisation with in-hospital and usually drug-resistant bacterial flora predisposing to surgical site infections.

The functional outcome after hip fracture is compromised, and usually there is a decline in physical activity, with almost half of the patients needing to use walking aids (Thomas et al., 2010; Dyer et al., 2016). On top of this decline, the presence of infection further decreases in a clinically significant way the physical activity of patients. The median Barthel index of 14 means that these patients had severe restrictions in mobility and self-care. Few studies report on the functional outcome after infection of hip fracture management. Partanen et al. (2006) reported that patients with infection had worse walking ability and more frequently used walking aids at 4 months but did not use a validated instrument. In the present study we showed that infection predisposed to worse clinical and functional results as demonstrated by the decline in Barthel score. Moreover, besides pre-fracture functional status, which has already been recorded, our study showed that infection independently affects the physical status of patients.

The Parker mobility score showed a trend (p=0.071) of being lower in patients with infections. Also, quality of life was poorer but without reaching the clinically significant difference of 0.08 or a statistical difference. A recent study disclosed a EQ-5D-5L index of 0.76 in patients with FRI of long bones, which is higher than our FRI group, probably because of the younger mean age of their cohort (60 vs. 79 years) (Walter et al., 2021). Finally, pain did not differ between groups. The reason for the discrepancy that patients complicated with infection are not experiencing worse quality of life while having worse physical function is not known.

Limitations of the study include the small number of patients. FRI after hip fracture is a rare event making data collection and analysis very difficult. In the literature, similar numbers of patients are reported, although the observation period varies from 2 to 10 years. Duckworth et al. (2012) reported on 43 patients and Edwards et al. (2008) on 41 patients in studies with the biggest numbers of patients. Also, the patients were not matched for the year of hip fracture. The control group was consecutive patients with hip fractures operated from January to June 2018 and was the best available regarding data collection. Another limitation is the fact that some cases of infection might receive treatment in another hospital. However, this seems a remote possibility as our unit is the reference centre for infections. Finally, as an observational study, confounding factors may exist. We tried to eliminate this by exploring possible confounding factors like ASA score and use of anticoagulants. Variables like steroid use or BMI was not available for all patients and were not included in the analysis. The strength of the study is that a validated instrument for assessment was used and that prospectively collected data were available.

## 5 Conclusions

Although the incidence of post-operative infection after hip fracture surgery is low, it increases mortality and imposes a significant limitation on the functional outcome. The delay to surgery increases the likelihood for infection. Therefore all physicians involved in the management of elderly with hip fractures should collaborate to develop optimisation pathways of these patients, which would allow early surgical management to mitigate the infection risk and the related consequences on the function, the mortality and the health care costs.

## Data Availability

Data are accessible from the electronic database of our department. Data are available from the corresponding author upon request.
